# Clinical Characteristics and Eosinophils in Young SARS-CoV-2-Positive Chinese Travelers Returning to Shanghai

**DOI:** 10.3389/fpubh.2020.00368

**Published:** 2020-07-10

**Authors:** Juan Yang, Xiaohui Zhao, Xueyuan Liu, Wanju Sun, Longzhu Zhou, Yongbing Wang, Haijing Sui

**Affiliations:** ^1^Department of Neurology, Shanghai Pudong New Area People's Hospital Affiliated to Shanghai University of Medicine & Health Sciences, Shanghai, China; ^2^Department of Neurology, Shanghai Tenth People's Hospital Affiliated to Tongji University School of Medicine, Shanghai, China; ^3^Shanghai Pudong New Area People's Hospital Affiliated to Shanghai University of Medicine & Health Sciences, Shanghai, China; ^4^Department of Image, Shanghai Pudong New Area People's Hospital Affiliated to Shanghai University of Medicine & Health Sciences, Shanghai, China

**Keywords:** COVID-19, SARS-CoV-2, viral pneumonia, clinical features, computed tomography, blood index

## Abstract

**Background:** The COVID-19 outbreak, which was first reported in Wuhan, China, in December 2019, began to spread throughout the world, and now involves over 200 countries.

**Methods:** A total of 37 overseas young and middle-aged people, who tested as SARS-CoV-2 positive upon their return to Shanghai, were enrolled for an analysis of their clinical symptoms, blood routine indexes, and lung CT images.

**Results:** The clinical symptoms were characterized by fever (51.4%), dry cough (13.5%), expectoration (27.0%), hypodynamia (21.6%), pharyngalia (10.8%), pharynoxerosis (8.1%), rhinobyon (13.5%), rhinorrhea (8.1%), muscular soreness (16.2%), and diarrhea (2.7%). In 16.2% of cases, no symptoms were reported. Fever was the most common symptom (51.40%). The pneumonic changes referred to the latticed ground glass imaging and similar white lung imaging accompanied by consolidated shadows. The rate of pneumonia was high (81.10%). We found that the exclusive percent of eosinophils was abnormally low. By analyzing the correlation of eosinophils, fever, and pneumonia, we found that the percentage of eosinophils was low in the COVID-19 patients afflicted with fever or pneumonia (*P* < 0.01). Additionally, pneumonia and fever were negatively correlated with the percentage of eosinophils and eosinophils/neutrophils ratio (*P* < 0.01, respectively), but not associated with pneumonia severity (*P* > 0.05). Fever was not correlated with pneumonia (*P* > 0.05).

**Conclusion:** A low percentage of eosinophils may be considered as a biomarker of pneumonia of COVID-19, but not as a biomarker of pneumonia severity.

## Introduction

The outbreak of COVID-19, officially termed by the World Health Organization (WHO) on 11 February, 2020 (1), was first reported in Wuhan, China, in December 2019. It has now been reported throughout the world, threatening nearly 200 countries. The novel coronavirus can cause severe pneumonia and acute respiratory distress syndrome (2). Moreover, it can induce vascular inflammation, myocarditis, and cardiac arrhythmias (3). This is a disease with a high fatality rate. Previous investigations have reported on the virus' route of transmission and its control measures, including the rapid diagnosis and immediate isolation of the victims, rigorous tracking, social distancing, and precautionary self-isolation (4–6). Some studies were quite concerned about the particular therapy (7–9) and the correlation of the clinical characteristics and lab indexes with the risk of death (10). Additionally, many others focused on the characteristics of CT imaging, but their focus was often placed on the comparison between pre- and post-treatment or on the analysis of the characteristics of lung computerized tomography (CT) in patients with different severities (11–13). In fact, the importance of early identification cannot be overemphasized; therefore, it is imperative that the early symptoms, lab indexes, and lung CT imaging of the young and middle-aged patients be better understood, especially the interrelation of early clinical symptoms with lab indexes and lung CT imaging.

As the global outbreak of COVID-19 intensified, a growing number of overseas Chinese nationals became anxious, longing for return to their own country. Working for Shanghai Pudong New Area People's Hospital, a hospital which is close to Shanghai Pudong International Airport, our doctors had the privilege of carrying out the mission of screening for the potential carriers of the novel coronavirus so that we could conduct their prompt isolation and proper treatment in our hospital.

By analyzing their clinical symptoms, blood routine indexes, and CT imaging, we came to summarize the typical characteristics in the current study. Since the returnees who tested positive were all either young or middle-aged without any disease history, the characteristics of the clinical symptoms and the changes of blood routine indexes and lung CT imaging could be typically different from those of other age groups. We believed that the current study could help the world better understand the clinical characteristics of the novel coronavirus in a particular age group.

## Materials and Methods

### Ethics Statement

This study was approved by the Medical Ethics Committee of Shanghai Pudong New Area People's Hospital, Shanghai, China. Written informed consent was obtained from all participants or their legally acceptable representatives.

### Patient Registration and Medical Examination

All overseas returnees, whose nasopharyngeal swab assays tested positive for SARS-CoV-2 by PCR at Shanghai Pudong New Area People's Hospital, were enrolled, the period of which had a time range of 1–23 March, 2020. As required by the National Health Commission, we performed the uniformed screening for the coronavirus as follows: (1) Ask for any travel history to or from the epidemic area(s); (2) Ask for symptoms such as a fever, dry cough, expectoration, pharyngalia, pharynoxerosis, rhinobyon, rhinorrhea, hypodynamic, muscular soreness, and diarrhea; (3) Perform a routine analysis of blood tests pertaining to hemoglobin, red blood cell count, hematocrit, mean corpuscular volume, average hemoglobin content, red cell distribution width, blood platelet count, thrombocytocrit, platelet distribution width, mean platelet volume, neutrophils percentage, eosinophils percentage, basophilic granulocyte percentage, monocyte percentage, and C-reactive protein; and (4) Administer CT to scan the lungs. We performed PCR assays to test the viral nucleic acids of SARS-CoV-2, used CT of SOMATOM Definition Flash (Germany), and performed a routine analysis of blood tests using Sysmex XT-4000i (Japan).

### Statistical Analysis

Statistical Package for the Social Sciences version 19.0 (SPSS Inc., Chicago, IL) was applied to the current statistical analysis; descriptive statistics were used to calculate by percentage the clinical characteristics of the infected returnees; chi-squared test was used to assess the rate differences; and *t*-test was used to assess the differences of age and lymphocyte between fever and no fever. The analyses of binary regression and spearman correlation were used to assess the correlation of eosinophils percentage, fever, and pneumonia. Linear regression was used to assess the correlation of four kinds of blood cells with fever and pneumonia severity.

## Results

### Clinical Symptoms

**Figure 1:**
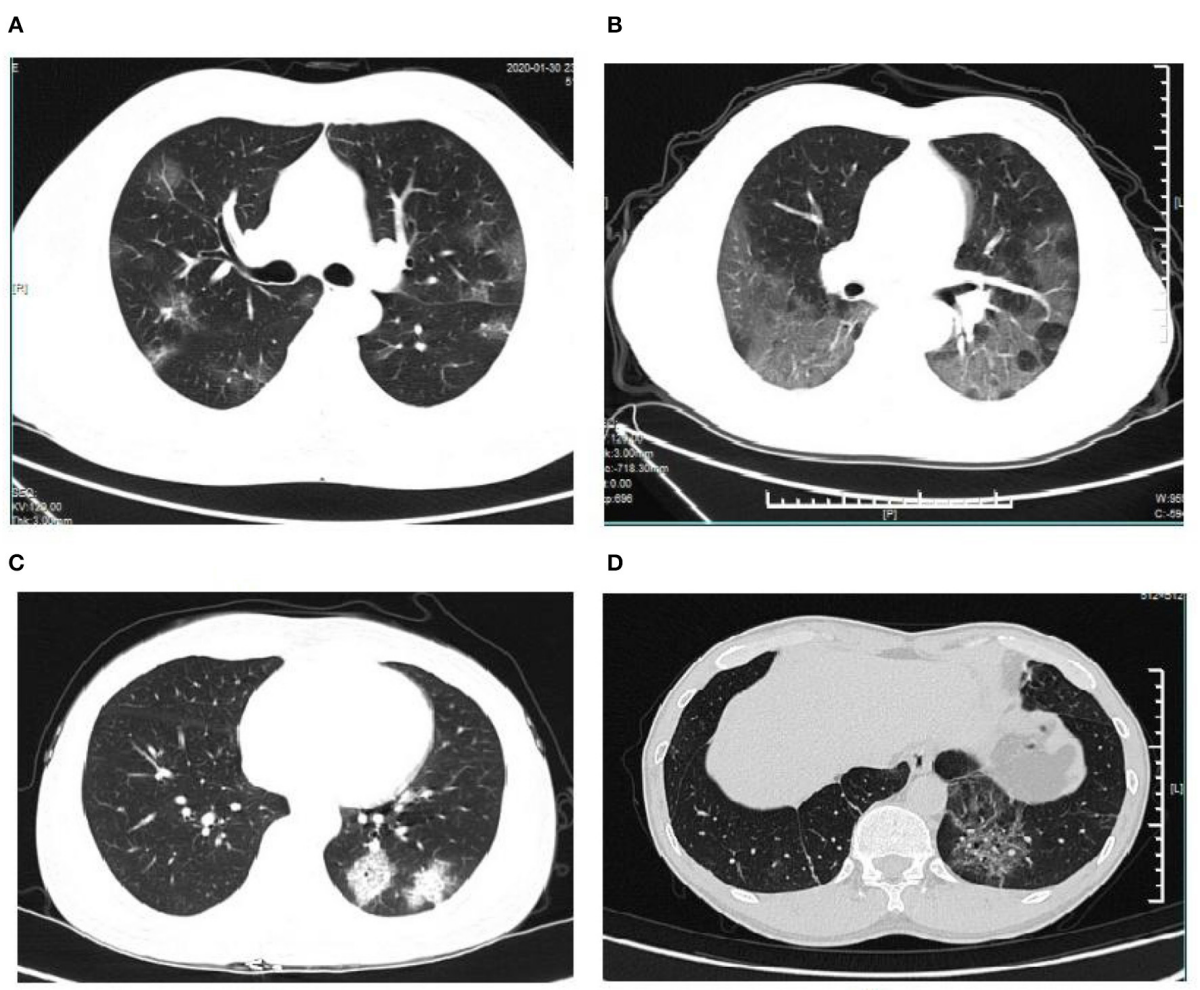
CT-signs of pneumonia in COVID-19 patients. **(A)** Latticed ground glass sign; **(B)** white-lung sign; **(C)** roadstone-like sign; **(D)** vascular cluster sign.

A total of 37 overseas returnees who were diagnosed with COVID-19 were recruited for the current study. The returnees were composed of overseas students and employees working in Spain, Italy, UK, France, and Dubai, who without exception were of Chinese nationality. Their age ranged from 19 to 67. Nineteen of the returnees complained of fever, and eight of them reported close contact with those who were confirmed positive. As indicated in Table 1, the base characteristics and clinical symptoms were acquired as the results, fever being the most common symptom (51.40%); as to the blood routine indexes, WBC and lymphocyte count were not recorded as “up or down,” but as what they were, while the others were recorded as “up or down.” PDW percentage and MPV and eosinophils percentage were significantly changed; their rates were 89.20, 81.10, and 45.90, respectively. Furthermore, eosinophils percentage was observed to be down as a single change, but when using other indexes, the changes reported were not unique. Without exception, the returnees received a lung CT scan, from which the pneumonic changes were characterized by four signs: the latticed ground glass sign, white-lung sign, roadstone-like sign, and vascular cluster sign (Figure 1). The number of lung lesions ranged from 1 to 41, which suggested different degrees of pneumonia severity. Each of the five lobes, three of the right, and two of the left, was affected by the coronavirus, with the margin damaged already. The rate of pneumonia was significantly high (81.10%).

**Table 1:** The base characteristics and clinical symptoms of positive COVID-19 participants.

	**n/mean$\pm \bar{x}$**	**%**
**Base characters**		
Age (year)	32.32 ± 15.71	
Duration of symptom (day)	3.50 ± 1.03	
Male	24	64.90%
**Clinical symptoms**		
Fever (Y)	19	51.40%^*^
Dry cough (Y)	5	13.50%
Expectoration (Y)	10	27.00%
Pharyngalia (Y)	4	10.80%
Pharynoxerosis (Y)	3	8.10%
Rhinobyon (Y)	5	13.50%
Running nose (Y)	3	8.10%
Hypodynamic (Y)	8	21.60%
Muscular soreness (Y)	6	16.20%
Diarrhea (Y)	1	2.70%
No symptom	6	16.20%
**Blood routine index**		%
PDW percentage		
Up	2	5.40%
Down	33	89.20%^*^
MPV (fl)		
Up	1	2.7%
Down	30	81.10%^*^
**Eosinophils percentage**		
Up	0^*^	0
Down	17	45.90%^*^
**Lung CT imaging**		
Pneumonia	30	81.10%^*^
Bilateral lesion	15	40.50%
Superior lobe lesion	13	35.10%
Mid-lobe lesion	7	18.90%
Inferior lobe	20	54.10%
Over-one-lobe lesion	14	37.80%
With pulmonary nodule	8	21.60%
Over-one lesion	16	43.20%
Over-ten lesion	8	21.60%
White-lung imaging	2	5.40%
No lesion	10	27.02%

(Y), positive; in one case over-one symptom occurred, thus adding up all numbers not equal to 37; Over-one lobe lesion, lesion over one pulmonary lobe; over-one lesion, the number of pulmonary lesion over one; over-ten lesion, the number of pulmonary lesion over ten; superior lobe lesion, the superior lobe affected; mid-lobe lesion, the mid-lobe affected; inferior lobe lesion, the inferior lobe affected.

* *Statistically significant*.

### The Correlative Analysis of Eosinophils Percentage, Fever, and Pneumonia

As indicated in Table 2, we made a correlative analysis of eosinophils percentage, fever, and pneumonia using three methods of statistical analysis. As fever was the most common symptom, all the returnees were subdivided into a fever group and no fever group; as rate of pneumonia was significantly high, all the returnees were subdivided into a pneumonia group and a no pneumonia group. The results of the Chi-squared test showed that between the two groups we analyzed the differences of the clinical symptoms, blood routine indexes, and lung CT images. As one of the blood routine indexes, eosinophils percentage was lower in those who had a fever than in those who did not (*P* < 0.05), and it was lower in the group with pneumonia than in the group without (*P* < 0.01). As indicated by the other indexes, no significant difference was observed between the fever and no fever group, and neither was it between the pneumonia and no pneumonia group (*P* > 0.05).

**Table 2:** The correlative analysis of eosinophils percentage, fever, and pneumonia.

		***P***	**x^**2**^**		
**Chi-squared test**	Eosinophils% (down) - Fever	0.03^⋆^	4.65		
	Lymphocytes count - Fever	0.34	1.89		
	Lymphocytes% - Fever	0.09	4.85		
	Neutrophils count-Fever	0.48	1.08		
	Neutrophils% - Fever	1.00	0.97		
	WBC count - Fever	0.48	2.00		
	Eosinophils% (down) - Pneumonia	0.008^*^	7.33		
	Lymphocytes count - Pneumonia	0.56	1.34		
	Lymphocytes% - Pneumonia	0.43	2.38		
	Neutrophils count - Pneumonia	1.00	0.24		
	Neutrophils% - Pneumonia	1.00	0.24		
	WBC count - Pneumonia	1.00	0.49		
**Spearman correlation analysis**		***P***	***r***		
	Eosinophils (%) - Pneumonia	0.006^*^	−0.44		
	Eosinophils (%) - Fever	0.03^⋆^	−0.35		
	Pneumonia - Fever	0.19	0.22		
	Lymphocytes count - Pneumonia	0.02^⋆^	0.38		
	Lymphocytes count - Fever	0.85	0.03		
	Lymphocytes% - Pneumonia	0.005^*^	0.45		
	Lymphocytes% - Fever	0.41	0.13		
	Neutrophils count - Pneumonia	0.67	−0.30		
	Neutrophils count - Fever	0.84	0.03		
	Neutrophils% - Pneumonia	0.02^⋆^	−0.38		
	Neutrophils% - Fever	0.91	−0.01		
	WBC count - Pneumonia	0.66	−0.07		
	WBC count - Fever	0.51	0.11		
**Binary regression analysis**		***P***	**OR**	**95%CI of OR**	
	Eosinophils (%) - Fever	0.03^⋆^	0.47	0.23	0.94
	Lymphocytes count - Fever	0.68	1.23	0.43	3.48
	Lymphocytes% - Fever	0.05	1.06	0.99	1.14
	Neutrophils count - Fever	0.76	0.91	0.52	1.61
	Neutrophils% - Fever	0.80	0.99	0.93	1.05
	WBC count - Fever	0.28	0.77	0.48	1.23
	Eosinophils (%) - Pneumonia	0.10	1.31	0.94	1.82
	Lymphocytes count - Pneumonia	0.35	1.87	0.49	7.07
	Lymphocytes% - Pneumonia	0.09	1.07	0.98	1.18
	Neutrophils count - Pneumonia	0.53	0.79	0.37	1.66
	Neutrophils% - Pneumonia	0.45	0.97	0.89	1.04
	WBC count - Pneumonia	0.40	1.28	0.71	2.33

⋆ P < 0.05;

* *P < 0.01*.

The results of Spearman correlation analysis showed that pneumonia is negatively correlated with the eosinophils percentage (*P* < 0.01); fever is negatively correlated with eosinophils (*P* < 0.01), but it was not correlated with pneumonia (*P* > 0.05). Moreover, lymphocyte count and lymphocyte percentage were positively correlated with pneumonia (*P* = 0.02, *P* = 0.005), and the neutrophils percentage was negatively correlated with pneumonia (*P* < 0.05), while the neutrophils count was not correlated with pneumonia (*P* > 0.05).

As indicated by the results of the Binary regression analysis, fever was negatively correlated with eosinophils percentage (*P* < 0.05), but pneumonia was not (*P* > 0.05). Additionally, the results of the Binary regression analysis demonstrated that the *P*-value in the correlation of lymphocytes with fever was low (*P* = 0.05), but as indicated by the Confidence intervals, it was not significant enough. The other blood routine indexes were not correlated with fever or with pneumonia (*P* > 0.05).

The results of Linear regression analysis showed that fever was negatively correlated with eosinophils percentage and eosinophils/neutrophils ratio (*P* < 0.05), but not with pneumonia severity (*P* > 0.05). The eosinophils percentage, eosinophils/neutrophils ratio, lymphocytes percentage, and neutrophils percentage were not associated with pneumonia severity, either (*P* > 0.05) (as indicated in Table 3).

**Table 3:** The correlation of fever and Pneumonia severity and four indexes (Linear regression).

		**B**	***P***	**95%CI of B**	
**Fever** **°C**					
	Eosinophils%	−0.81	0.03^⋆^	−0.88	1.54
	Lymphocytes%	−0.01	0.56	−0.04	0.02
	Neutrophils%	−0.03	0.10	−0.07	0.007
	Eosinophils/Neutrophils	−48.34	0.01^⋆^	−87.91	−8.77
	ratio				
	Pneumonia severity	0.006	0.51	−0.01	0.02
**Pneumonia severity**					
	Eosinophils%	−0.08	0.99	−15.68	15.52
	Lymphocytes%	−0.26	0.42	−0.94	0.41
	Neutrophils%	−0.57	0.14	−1.35	0.21
	Eosinophils/Neutrophils	−27.43	0.94	−885.98	831.12
	ratio				
	Fever°C	2.36	0.51	−5.05	9.79

⋆ *P < 0.05*.

All the detailed data analysis are available as six tables in the Supplementary Materials.

## Discussions

In the current study, we found that the young and middle-aged returnees who tested positive for SARS-CoV-2 had symptoms including fever, dry cough, expectoration, pharyngalia, pharynoxerosis, rhinobyon, rhinorrhea, hypodynamic, muscular soreness, and diarrhea. Fever was clearly the most common symptom. One previous study reported that the clinical symptoms were fever (57.1%), cough (35.7%), chest tightness/pain (21.4%), fatigue (21.4%), and sore throat (7.1%) (14), which coincided with our findings of fever (51.4%), dry cough (13.5%), expectoration (27.0%), hypodynamic (21.6%), and pharyngalia (10.8%). Moreover, we observed the clinical symptoms of pharynoxerosis (8.1%), rhinobyon (13.5%), rhinorrhea (8.1%), muscular soreness (16.2%), and diarrhea (2.7%).

Moreover, we found that the young and middle-aged returnees with COVID-19 had abnormal blood routine indexes. The eosinophils percentage was observed to be down as a single change, but using the other indexes they were observed to be up or down, the changes of which were not unique. This finding indicated that eosinophils percentage may serve as an important biomarker of COVID-19. The evidence that WBC was down or normal and lymphocyte percentage was down had been confirmed in one of the previous investigations that reported that nearly 70% of the patients had normal or decreased white blood cell counts (71.4 vs. 28.6%), and that 50% had lymphocytopenia (14). But we came to realize that a low percentage of eosinophils could be correlated with COVID-19.

We also found that the rate of pneumonia was significantly high (81.10%), with such pneumonic changes as latticed ground glass imaging, white-lung imaging, and consolidated shadow. It was previously reported that COVID-19 individuals would present with typical ground-grass opacities and other CT features (15), which coincided with our findings.

In the case of COVID-19, the most common symptom was fever; the eosinophils percentage was significantly low, and the rate of pneumonia was high, as indicated by lung CT. Thus, we examined whether there existed an inherent correlation among the three evidences. We used a Chi-squared test to assess the different percentages of eosinophils between the patients with fever and those without, and between the patients with pneumonia and those without. We used Spearman correlation analysis and binary regression analysis to examine the correlation of eosinophils percentage, fever, and pneumonia.

The differences in clinical symptoms, blood routine index, and lung CT imaging between the COVID-19 patients with a fever and without showed that the percentage of eosinophils was lower in those who had a fever than in those who did not. These findings indicated that the low percentage of eosinophils was correlated with a fever in COVID-19 patients. As indicated by the differences in clinical symptom and blood routine index between the patients with pneumonia and those without, the percentage of eosinophils was low in the COVID-19 patients afflicted with pneumonia. These findings indicated that the low percentage of eosinophils could be correlated with pneumonia in COVID-19 patients.

As indicated by the results of the Spearman correlation analysis, a significant correlation of low eosinophils percentage was observed with fever and with pneumonia, while no correlation was found between fever and pneumonia. These findings verified the conclusion we made based on the results of the Chi-squared test and Linear regression. Furthermore, the results of the Binary regression analysis suggested that fever was negatively correlated with the percentage of eosinophils. Although the Binary regression analysis demonstrated that pneumonia was not correlated with the percentage of eosinophils, we considered that the small-sized data of this study could have affected the result. Although it was not statistically significant enough, this finding was clinically significant enough to be taken seriously, because pneumonia was negatively correlated with the percentage of eosinophils, as indicated in the results of the Chi-squared test and Spearman correlation analysis. The Spearman correlation analysis also showed that lymphocytes count and percentage were positively correlated with pneumonia (*P* = 0.02, *P* = 0.005); the percentage of neutrophils was also negatively correlated with pneumonia (*P* < 0.05), while the count of neutrophils was not correlated with pneumonia (*P* > 0.05). The different *P*-value suggested that the percentage of lymphocytes and neutrophils was more sensitive than the count of lymphocytes and neutrophils. Furthermore, low eosinophils percentage, high lymphocytes percentage, and low neutrophils percentage could be biomarkers of pneumonia as well, although this was not supported by the Chi-squared test. Additionally, the results of the Binary regression analysis demonstrated that the *P*-value in the correlation of lymphocytes with fever was low, but as indicated by the confidence intervals, it was not significant enough. Therefore, we believe that the proof of lymphocytes correlating with fever was not absolute.

From the results of the interrelation of the clinical symptoms and Lung CT imaging with the percentage of eosinophils, we found that COVID-19 patients with a low percentage of eosinophils were likely to develop a fever and pneumonia, which indicated an interrelation between both. Our findings had been supported by the previously reported studies in terms of eosinophils percentage in COVID-19 (16). Eosinophils percentage was considered to be a pro-inflammatory factor, playing a pleotropic role as regulatory cells involved in protective immunity, including antiviral responses and diverse physiological responses (17). All these functions could explain why a low percentage of eosinophils meant a patient was more likely to develop a fever and pneumonia in our study. Furthermore, another study concluded that SARS-CoV-2 vaccine candidates might induce eosinophil-associated disease (18), which also supported our conclusion. A previous meta-analysis pooled three studies, which covered 294 patients, 25.5% of whom had severe COVID-19, the conclusion of which suggested that eosinopenia may not be associated with an unfavorable progression of COVID-19 (19). This was in line with our conclusion that low percentage of eosinophils may be considered as a biomarker of pneumonia due to COVID-19, but not as a biomarker of pneumonia severity. Further verification merits a larger number of samples for investigation and analysis.

## Conclusion

By investigating the young and middle-aged returnees infected with SARS-CoV-2, we acquired a deep insight into the correlation among fever, low eosinophils percentage, and pneumonia. Our findings indicated that a correlation was observed between eosinophils percentage and fever and between low eosinophils percentage and pneumonia, and that no correlation was observed between fever and pneumonia. With a low percentage of eosinophils, fever and pneumonia were more likely to develop in COVID-19 patients. Thus, a low percentage of eosinophils could serve as a biomarker of COVID-19 pneumonia, but not as a biomarker of pneumonia severity.

## Limitation

This was a small sample study, which, to a great extent, was decided by the context in which we conducted the investigation. The viral load was not tested; the test result of SARS-CoV-2 was just recorded as negative or positive.

## Data Availability Statement

The datasets presented in this study can be found in online repositories. The names of the repository/repositories and accession number(s) can be found in the article/Supplementary Material.

## Ethics Statement

The studies involving human participants were reviewed and approved by the Medical Ethics Committee of Shanghai Pudong New Area People's Hospital, Shanghai, China. The patients/participants provided their written informed consent to participate in this study.

## Author Contributions

JY and HS conceived and designed the project. HS performed all the experiments and prepared the figures. JY performed all the experiment and wrote the manuscript. All authors reviewed the manuscript.

## Conflict of Interest

The authors declare that the research was conducted in the absence of any commercial or financial relationships that could be construed as a potential conflict of interest.
