# Supplementary material for: Clinical Characteristics and Eosinophils in Young SARS-CoV-2-Positive Chinese Travelers Returning to Shanghai
Source: Front Public Health. 2020 Jul 10;8:368. doi: 10.3389/fpubh.2020.00368 (PMC7365885; doi:10.3389/fpubh.2020.00368)
Supplement: Supplementary file 1 [file Data_Sheet_1.docx]

**Table 1The base characters and clinical symptoms of the positive**

|  | **n/mean±‾x** | % |
| --- | --- | --- |
| **Base characters** |  |  |
| Age(year) | 32.32±15.71 |  |
| Duration of symptom (day) | 3.50±1.03 |  |
| Male | 24 | 64.90% |
| **Clinical symptoms** |  |  |
| Fever (Y) | 19 | 51.40%* |
| Dry cough (Y) | 5 | 13.50% |
| Expectoration(Y) | 10 | 27.00% |
| Pharyngalia (Y) | 4 | 10.80% |
| Pharynoxerosis (Y) | 3 | 8.10% |
| Rhinobyon (Y) | 5 | 13.50% |
| Running nose (Y) | 3 | 8.10% |
| Hypodynamic (Y) | 8 | 21.60% |
| Muscular soreness (Y) | 6 | 16.20% |
| Diarrhea (Y) | 1 | 2.70% |
| No symptom | 6 | 16.20% |
| Source |  |  |
| Spain | 6 | 16.20% |
| UK | 7 | 18.91% |
| Italy | 13 | 35.13% |
| France | 6 | 16.20% |
| Dubai | 5 | 13.50% |

(Y) = positive; in one case over-one symptom occurred, thus adding up all numbers not equal to 37; *statistically significant

**Table 2** The characters of blood routine analysis of the positive

| **Blood routine index** | **n/mean±‾x** | % |
| --- | --- | --- |
| WBC(10^9/L) | 5.64**±**1.45 |  |
| Percent of [Lymphocyte](file:///C:\Users\yangjuan\Desktop\newcoronavirus\javascript:;) | 30.5**±**10.92 |  |
| **MCV(fl)** |  |  |
| up | 1 | 2.70% |
| down | 7 | 18.90% |
| **MCH(pg)** |  |  |
| up | 4 | 10.80% |
| down | 9 | 24.30% |
| Percent of **RDW** |  |  |
| up | 11 | 29.70% |
| down | 0 | 0 |
| Percent of **PDW** |  |  |
| up | 2 | 5.40% |
| down | 33 | 89.20%* |
| **MPV(fl)** |  |  |
| up | 1 | 2.7% |
| down | 30 | 81.10%* |
| Percent of m**onocyte** |  |  |
| up | 6 | 16.20% |
| down | 0 | 0 |
| **Blood platelet count**(10^9/L) |  |  |
| up | 1 | 2.7% |
| down | 0 | 0 |
| Percent of t**hrombocytocrit** |  |  |
| up | 1 | 2.70% |
| down | 1 | 2.70% |
| Percent of e**osnophils** |  |  |
| up | 0* | 0 |
| down | 17 | 45.90%* |
| **CRP(mg/L)** |  |  |
| up | 4 | 10.80% |
| down | 0 | 0 |

*Statistically significant

**Table 3** The characters of lung CT imaging of the positive

| **Lung CT imaging** | n | % |
| --- | --- | --- |
| Pneumonia | 30 | 81.10% |
| Bilateral lesion | 15 | 40.50% |
| Superior lobe lesion | 13 | 35.10% |
| Mid-lobe lesion | 7 | 18.90% |
| Inferior lobe | 20 | 54.10% |
| Over-one-lobe lesion | 14 | 37.80% |
| With pulmonary nodule | 8 | 21.60% |
| Over-one lesion | 16 | 43.20% |
| Over-ten lesion | 8 | 21.60% |
| White-lung imaging | 2 | 5.40% |
| No lesion | 10 | 27.02% |

**Table 4** The differences of clinical symptom, blood routine index and lung CT imaging between the positive with fever and the positive without

| Variable | Fever (N=19) | No-fever (N=18) | t/x2 | P |
| --- | --- | --- | --- | --- |
| **Clinic Symptom** |  |  |  |  |
| Expectoration (Y) | 9(90.0%) | 1(10.0%) | 8.19 | 0.005★ |
| Pharyngalia (Y) | 2(50.0%) | 2(50.0%) | 0.003 | 0.67 |
| Pharynoxerosis(Y) | 1(33.3%) | 2(66.7%) | 0.42 | 0.47 |
| Rhinobyon (Y) | 2(40.0%) | 3(60.0%) | 0.29 | 0.47 |
| Running nose (Y) | 2(66.7%) | 1(33.3%) | 0.31 | 0.52 |
| Hypodynamic (Y) | 4(50.0%) | 4(50.0%) | 0.007 | 0.62 |
| Muscular soreness (Y) | 4(66.7%) | 2(33.3%) | 0.67 | 0.35 |
| **Blood routine index** |  |  |  |  |
| WBC | 5.88**±**1.58 | 5.38**±**1.29 | 1.06 | 0.29 |
| Percent of [lymphocyte](javascript:;) | 27.04**±**11.50 | 34.15**±**9.23 | -2.06 | 0.04 |
| MCV down | 3(42.9%) | 4(57.1%) | 1.42 | 0.49 |
| MCH down | 5(55.6%) | 4(44.4%) | 1.25 | 0.53 |
| RDW up | 7(63.7%) | 4(36.4%) | 0.94 | 0.27 |
| PDW down | 15(45.5%) | 18(54.5%) | 4.24 | 0.12 |
| MPV down | 15(50.0%) | 15(50.0%) | 0.97 | 0.61 |
| Percent of monocyte up | 4(66.7%) | 2 (33.3%) | 0.67 | 0.35 |
| Blood platelet count down | 1(100%) | 0(0.0%) | 0.97 | 0.51 |
| Thrombocytocrit down | 1(100%) | 0(0%) | 2.00 | 0.36 |
| Percent of eosnophils down | 12(70.6%) | 5(29.4%) | 4.65 | 0.03★ |
| CRP up | 2 (50.0%) | 2 (50.0%) | 0.003 | 0.67 |
| **Lung CT imaging** |  |  |  |  |
| Pneumonic change | 17(56.7%) | 13 (72.2%) | 1.79 | 0.18 |
| Bilateral lesion | 10(66.7%) | 5 (33.3%) | 2.99 | 0.22 |
| Superior lobe lesion | 9(69.2%) | 4(30.8%) | 3.89 | 0.14 |
| Mid-lobe lesion | 5(71.4%) | 2 (28.6%) | 1.54 | 0.46 |
| Inferior lobe | 11(55.0%) | 9(45.0%) | 1.17 | 0.55 |
| Over-one lobe | 10(71.4%) | 4 (28.6%) | 6.12 | 0.04★ |
| Over-one lesion | 11(68.7%) | 5 (31.3%) | 8.29 | 0.01★ |
| Over-ten lesion | 4(50.0%) | 4(50.0%) | 0.04 | 0.98 |
| With pulmonary nodule | 3(37.5%) | 5(62.5%) | 0.78 | 0.37 |

**★**P<0.05;over-one lesion lobe = pulmonary lobe with over-onelesion; over-one lesion = the number of pulmonary lesion over one; over-ten lesion = the number of pulmonary lesion over ten

**Table 5** The differences of clinical symptoms and blood routine indexes between with- and without-pneumonia lung CT imaging

|  | Pneumonia | Normal | x^2^ | P |
| --- | --- | --- | --- | --- |
| **Symptoms** |  |  |  |  |
| Fever(Y) | 17(56.7%) | 2 (28.6%) | 1.79 | 0.18 |
| Dry cough(Y) | 5 (16.7%) | 0(0%) | 1.34 | 0.32 |
| Expectoration(Y) | 10 (33.3%) | 0 (0%) | 3.19 | 0.08 |
| Pharyngalgia(Y) | 4 (13.3%) | 0 (0%) | 1.04 | 0.41 |
| Pharyngoxerosis(Y) | 3 (10.0%) | 0 (0%) | 0.76 | 0.52 |
| Rhinobyon(Y) | 5(16.7%) | 0 (0%) | 1.34 | 0.32 |
| Running nose(Y) | 3 (10.0%) | 0 (0%) | 0.76 | 0.52 |
| Hypodynamic(Y) | 7 (23.3%) | 1 (14.3%) | 0.27 | 0.52 |
| Muscular soreness(Y) | 6(20.0%) | 0 (0%) | 1.67 | 0.25 |
| **Blood routine indexes** |  |  |  |  |
| MCV(up) | 1(3.3%) | 0 (0%) | 2.38 | 0.3 |
| (down) | 7(23.3%) | 0 (0%) |  |  |
| (normal) | 22(73.3%) | 7(100.0%) |  |  |
| MCH(up) | 4(13.3%) | 0 (0%) | 4.67 | 0.09 |
| (down) | 9(30.0%) | 0 (0%) |  |  |
| (normal) | 17(56.7%） | 7(100.0%) |  |  |
| RDW(up) | 19(63.3% | 7(100.0%) | 3.65 | 0.06 |
| (normal) | 11(36.7%) | 0 (0%) |  |  |
| PDW(UP) | 2(6.7%) | 0 (0%) | 1.04 | 0.59 |
| (down) | 26(86.7%) | 0 (0%) |  |  |
| (normal) | 2(6.7%) | 7(100.0%) |  |  |
| MPV(up) | 1(3.3%) | 0 (0%) | 2.01 | 0.36 |
| (down) | 23(76.7%) | 0 (0%) |  |  |
| (normal) | 6(20.0%) | 7(100.0%) |  |  |
| Percent of monocyte(up) | 6(20.0%) | 0 (0%) | 1.67 | 0.25 |
| (normal) | 24(80.0%) | 7(100.0%) |  |  |
| Blood platelet count(down) | 1(3.3%) | 0 (0%) | 0.24 | 0.81 |
| (normal) | 29(96.7%) | 7(100.0%) |  |  |
| Thrombocytocrit(up) | 1(3.3%) | 7(100.0%) | 0.49 | 0.78 |
| (down) | 1(3.3%) | 0 (0%) |  |  |
| (normal) | 28(93.3%) | 0 (0%) |  |  |
| Percent of eosnophils(down) | 17(56.7%） | 0 (0%) | 7.33 | 0.008* |
| (normal) | 13(43.3%) | 7(100.0%) |  |  |
| CRP(up) | 4(13.3%) | 0 (0%) | 1.04 | 0.41 |
| (normal) | 26(86.7%) | 7(100.0%) |  |  |

Note:*P<0.01

**Table 6** The differences of clinical symptoms and lung CT imaging between down- and normal-percent of eosnophils

|  | Percent of eosnophils(down) | Normal | x^2^ | P |
| --- | --- | --- | --- | --- |
| **Symptoms** |  |  |  |  |
| Fever(Y) | 12 (70.6%) | 7(35.0%) | 4.65 | 0.03★ |
| Dry cough(Y) | 3(17.6%) | 2 (10.0%) | 0.46 | 0.42 |
| Expectoration(Y) | 7(41.2%) | 3 (15.0%) | 3.19 | 0.07 |
| Pharyngalgia(Y) | 3 (17.6%) | 1 (5.0%) | 1.52 | 0.24 |
| Pharyngoxerosis(Y) | 2(11.8%) | 1 (5.0%) | 0.56 | 0.43 |
| Rhinobyon(Y) | 2(11.8%) | 3(15.0%) | 0.08 | 0.58 |
| Running nose(Y) | 1 (5.9%) | 2 (10.0%) | 0.20 | 0.56 |
| Hypodynamic(Y) | 4 (23.5%) | 4(20.0%) | 0.06 | 0.55 |
| Muscular soreness(Y) | 3 (17.6%) | 3(15.0%) | 0.04 | 0.58 |
| **Lung CT imaging** |  |  |  |  |
| Pneumonia | 17 (100%) | 13(65.0%) | 7.33 | 0.008* |
| Bilateral lesion | 8 (47.1%) | 7(35.0%) | 0.57 | 0.75 |
| Superior lobe lesion | 8(47.1%) | 5(25.0%) | 2.71 | 0.24 |
| Mid-lobe lesion | 5 (29.4%) | 2(10.0%) | 2.45 | 0.28 |
| Inferior lobe | 10(58.8%) | 10(50.0%) | 1.01 | 0.49 |
| Over-one lobe | 8 (47.1%) | 6(30.0%) | 1.58 | 0.44 |
| Over-one lesion | 9 (52.9%) | 7(35.0%) | 2.75 | 0.22 |
| Over-ten lesion | 5 (29.4%) | 3(15.0%) | 1.47 | 0.47 |
| With pulmonary nodule | 5(62.5%) | 3(37.5%) | 0.78 | 0.37 |

Note:★P<0.05,*P<0.01
